# Melanoma patient-derived xenografts accurately model the disease and develop fast enough to guide treatment decisions

**DOI:** 10.18632/oncotarget.2445

**Published:** 2014-09-08

**Authors:** Berglind O. Einarsdottir, Roger Olofsson Bagge, Joydeep Bhadury, Henrik Jespersen, Jan Mattsson, Lisa M. Nilsson, Katarina Truvé, Marcela Dávila López, Peter Naredi, Ola Nilsson, Ulrika Stierner, Lars Ny, Jonas A. Nilsson

**Affiliations:** ^1^ Department of Surgery, Institute of Clinical Sciences, Sahlgrenska Academy at the University of Gothenburg, Sahlgrenska University Hospital, Gothenburg, Sweden; ^2^ Department of Oncology, Institute of Clinical Sciences, Sahlgrenska Academy at the University of Gothenburg, Sahlgrenska University Hospital, Gothenburg, Sweden; ^3^ Department of Biomedicine, Institute of Biomedicine, Sahlgrenska Academy at the University of Gothenburg, Sahlgrenska University Hospital, Gothenburg, Sweden; ^4^ Sahlgrenska Translational Melanoma Group at the Sahlgrenska Cancer Center, Gothenburg, Sweden; ^5^ The Bioinformatics Core Facility at the University of Gothenburg, Gothenburg, Sweden

**Keywords:** melanoma, mouse models, patient-derived xenografts

## Abstract

The development of novel therapies against melanoma would benefit from individualized tumor models to ensure the rapid and accurate identification of biomarkers of therapy response. Previous studies have suggested that patient-derived xenografts (PDXes) could be useful. However, the utility of PDXes in guiding real-time treatment decisions has only been reported in anecdotal forms. Here tumor biopsies from patients with stage III and IV metastatic malignant melanoma were transplanted into immunocompromised mice to generate PDXes. 23/26 melanoma biopsies generated serially transplantable PDX models, and their histology, mutation status and expression profile resembled their corresponding patient biopsy. The potential treatment for one patient was revealed by an *in vitro* drug screen and treating PDXes with the MEK inhibitor trametinib. In another patient, the *BRAF* mutation predicted the response of both the patient and its corresponding PDXes to MAPK-targeted therapy. Importantly, in this unselected group of patients, the time from biopsy for generation of PDXes until death was significantly longer than the time required to reach the treatment phase of the PDXes. Thus, it could be clinically meaningful to use this type of platform for melanoma patients as a pre-selection tool in clinical trials.

## INTRODUCTION

Cutaneous malignant melanoma arises from melanocytes and is potentially curable with surgical excision of early, thin lesions. Therefore, prompt detection, diagnosis and adequate removal of such lesions are of the utmost importance. If the disease progresses undetected, it has a very poor prognosis, and stage IV melanoma has a 10-year survival of around 10% [1].

Somatic mutations during cutaneous melanoma development have been discovered. Mutations resulting in the activation of the MAPK pathway include *NRAS* mutations (20-25%; codon Q61) and *BRAF* (50-65%; codon V600). For *BRAF*-mutant tumors, impressive therapeutic responses have been achieved by the development of mutation-specific inhibitors [2, 3]. BRAF inhibitors like vemurafenib and dabrafenib improve both overall survival (OS) and progression-free survival (PFS), but relapse due to resistance eventually develops. Resistance can develop via a variety of mechanisms. However, because most mutations affect the same pathway, it is noteworthy that combination treatment with BRAF and MEK inhibitors has shown promising results in clinical trials [4].

The discovery of novel targeted and immune therapies has revolutionized melanoma treatment [5]. However, melanoma continues to have a poor prognosis. Unfortunately, when novel therapeutics are designed against proteins encoded by non-mutated genes, drug developers have a lack of biomarkers. To overcome this problem, we hypothesized that the tumor’s response to a treatment could serve as a biomarker for inclusion into clinical trials and for the pre-clinical discovery of useful biomarkers [6]. Patient-derived xenografts (PDXes), cell lines and genetic analyses of patients’ tumors are possible tools to enable biomarker discovery [7, 8]. A high degree of similarity has been demonstrated between PDXes and the corresponding tumor in the patient. Previous studies on melanoma PDXes have shown that they even can have predictive values in metastasis prognosis [9]. In some cases, the treatment of PDXes predicts treatment responses in patients [10]. Here, we describe a platform (Figure S1) used to characterize resected melanoma tumors *ex vivo* and *in vivo*. We found that the procedure yields data that can be translated to the clinic and demonstrate, for the first time, that this procedure is temporally feasible for the majority of melanoma patients at a University Hospital.

## RESULTS

This study aimed to generate a platform that can be used to direct the right patient to the right clinical trial or treatment. The results of the first thirty consecutively recruited patients with cutaneous melanoma included in the study at the Sahlgrenska University Hospital, Gothenburg, Sweden, are presented. As shown in Table 1, the melanomas were lymph node metastases (stage IIIC) or distant metastases (stage IV). Four were excluded; of these, one biopsy sample that grew in mice was shown to be of uveal origin, one sample which did not grow originated from a lymph node devoid of tumor cells according to the pathology report, and two samples were lost because of mouse accidents. Tumors from 23 out of the remaining 26 patients exhibited subcutaneous growth, in accordance with the published excellent take rate of cutaneous melanoma in the NOG mouse [11].

**Table 1:** Patient characteristics

Patient ID	Age	Sex	Clinical stage	Tumor site
M120511B	55	Male	IV	Subcutaneous
M120514	76	Female	IV	Subcutaneous
M120521A	61	Male	IV	Subcutaneous
M120521B	77	Female	IV	Subcutaneous
M120903	59	Male	IIIC	Lymph node
M120905	62	Male	IIIC	Lymph node
M120910B	81	Male	IV	Subcutaneous
M120913	78	Male	IV	Ascites
M121113	42	Female	IIIC	Lymph node
M121123	83	Female	IIIC	Lymph node
M121211	82	Male	IIIC	Lymph node
M121213A	70	Male	IIIC	Lymph node
M121218	67	Male	IV	Mesentery
M121221	55	Female	IV	Lymph node
M130111	54	Male	IIIC	Subcutaneous
M130116	39	Female	IV	Lymph node
M130128A	70	Male	IV	Subcutaneous
M130128B	75	Male	IV	Subcutaneous
M130204B	80	Male	IIIC	Lymph node
M130214	78	Male	IV	Subcutaneous
M130226	63	Female	IV	Subcutaneous
M130228	69	Male	IIIC	Subcutaneous
M130624	42	Female	IV	Spleen
M131004	66	Female	IV	Subcutaneous
M131118	41	Female	IIIC	Lymph node
M140117	75	Female	IV	Subcutaneous

**Figure 1:**
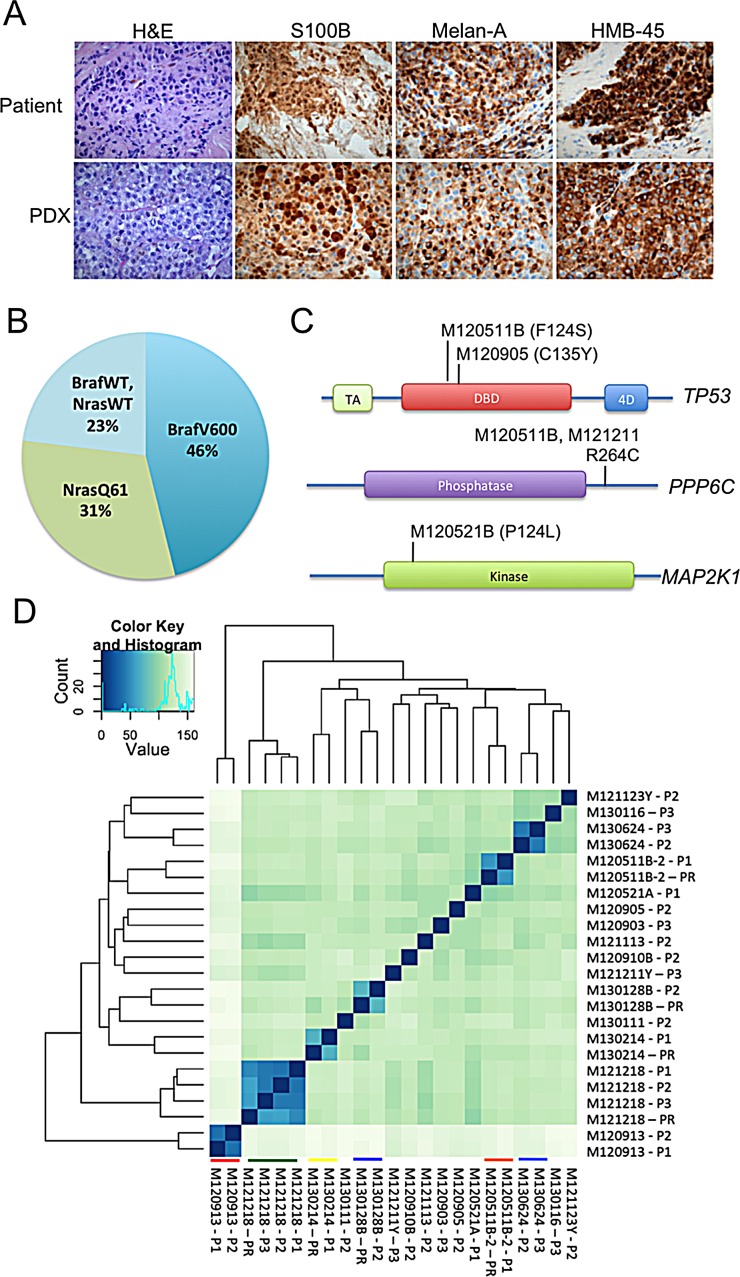
PDX models are similar to human melanoma in terms of histology, frequency and type of mutation, and their expression profiles (A) Representative images of H&E-stained formalin-fixed tumors from different PDX-models are shown. All samples were processed according to standard procedures at the clinical pathology lab. A clinical pathologist verified their similarity to human melanoma. (B) A pie chart of the mutation frequencies of the tumors engrafted is shown. Mutations determined by allele-specific PCR, exome/RNA-sequencing and Sanger sequencing were identical (where applicable). (C) Mutations were also found in genes previously identified to be mutated at a lower frequency in melanoma than the *BRAF*/*NRAS* genes. Illustrated here are mutations found in *TP53*, *MAP2K1* and *PPP6C* [26]. (D) The transcriptome of melanoma in patients and mice is highly similar. Unsupervised clustering analyses of RNAseq data generated from patient samples (PR) and tumors from PDXes (P1-P3) was performed using the DESeq2 package [27]. Samples originating from the same patient cluster together and are highlighted by colored lines. Heat-maps were generated showing the Euclidean distance between the samples calculated from the variance stabilized transformation [28].

PDX models are generally regarded as accurate models of human cancer [7, 8]. To validate this in our models, paraffin-embedded tumors were sectioned and analyzed by a board-certified clinical pathologist (O.N.). Stainings with H&E and antibodies against the highly characterized melanoma markers S100B, HMB-45A and Melan-A revealed the highly cellular growth pattern of melanoma, and resemblance to the tumor biopsies from patients was observed (Figure 1A and Figure S2A-B).

To further characterize the PDX models, we performed next-generation sequencing of exomes and/or the transcriptome (RNAseq). As shown in Table S1, Table S2 and Figure 1B, Most of the tumors had a *BRAF* V600 mutation or a *NRAS* Q61 mutation, well-known driver mutations in melanoma. Where applicable, perfect concordance was observed between the mutation statuses generated by NGS, allele-specific PCR and Sanger sequencing performed at the Hospital’s molecular pathology unit (Table S2). We also observed that some tumors exhibited mutations in the *TP53*, *PPP6C* or *MAP2K1* genes (Figure 1C) and that some tumors had a low amount of raw sequencing reads of the tumor suppressor gene *CDKN2A* (Table S1). These are known genetic alterations in melanoma, and our PDX models will be useful models to identify new therapies against these oncogenic lesions.

RNAseq data can also be used to generate expression profiles since the amount of sequencing reads is directly proportional to the gene expression. When comparing expression profiles from patient biopsies and PDXes by unsupervised hierarchical clustering we noted that samples originating from the same patient clustered together (Figure 1D). In one case, M121218, the high level of similarity in gene expression was maintained for three passages in mice. The data suggest that the melanoma cells growing in mice do not divert overtly from their original tumor in the patient.

**Figure 2:**
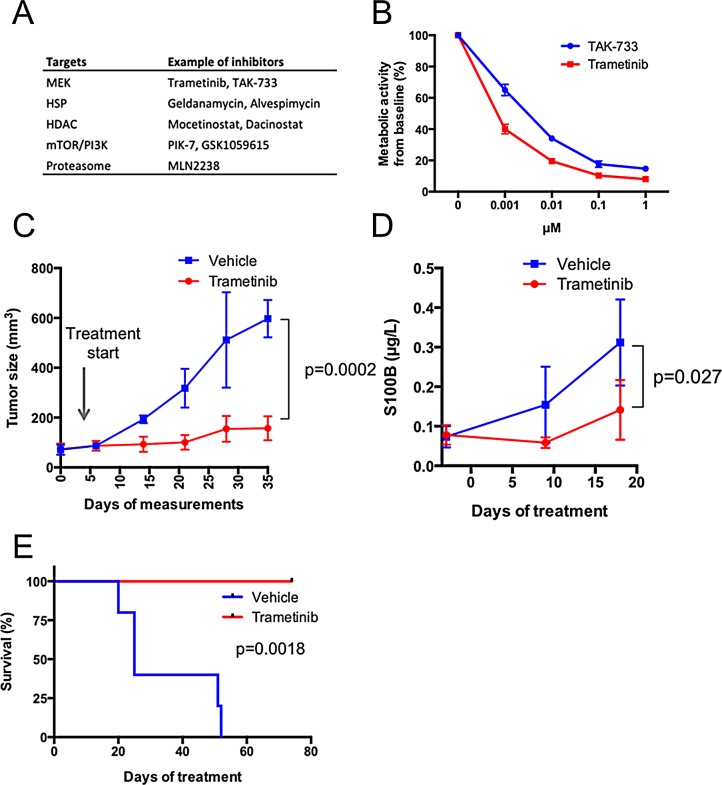
Melanoma cells from case 1 (patient M120903) respond to MEK inhibitors *in vitro* and *in vivo* (A) A dissected lymph node biopsy was used to establish xenografts and a cell culture on the day of lymphadenectomy. Less than one week after seeding, the cells were split into four 96-well plates, where each well contained 1 μM of one inhibitor or a DMSO control from a drug library of 319 compounds plus DMSO controls [6]. Three days later, Cell-Titer-Glo reagent (Promega) was added, and the metabolic activity (ATP production) were read in a luminometer. Shown here are the top hits of the screen, scored as compounds exhibiting a >80% reduction of viability. (B) Because two MEK inhibitors were identified in the screen, they were used in dose-response experiments measuring metabolic activity with Cell-Titer-Glo. Shown are mean values ± standard deviation. (C) Ten P3 PDXes were randomized to receive 0.3 mg/kg trametinib twice daily for five days a week by oral gavage (n=5) or vehicle (n=5). Tumor growth was measured with a caliper and mean tumor volumes was plotted from the time when the growth rate was highest ± standard deviation. Tumor growth pattern of individual mice is shown in Supplemental Figure S3B. (D) Blood samples were drawn before treatment initiation and at two time points after treatment initiation. Level of the melanoma marker S100B was measured in plasma by ELISA. E) Survival curve of the mice treated with vehicle or trametinib. The health status of the mice was determined by weight and tumor size. The ethics permit allowed a weight loss of <20% and a base length of maximum 10 mm. When these ethics limits were achieved (n=4) or the mice displayed serious signs of illness (n=1), the mice were sacrificed, and the time point was recorded.

To test different treatments—FDA-approved or in different phases of clinical development—we treated P3 PDX mice for at least 3 weeks with drugs and monitored the effects with caliper measurements of the individual tumors once a week. We also followed mice undergoing treatment using blood sampling and measurements of human S100B levels, a clinical biomarker of melanoma growth and progression. The first case description is of a patient who presented with lymph node metastasis. The resected material was dispersed and used to create a PDX and short-term cell cultures. The tumor cells grew very aggressively, enabling the *in vitro* drug screen and the development of a PDX (M120903). Before revealing that the tumor exhibited an *NRAS*^Q61^ mutation (Table S2 and Figure S3A), the drug screen suggested MAPK pathway engagement and cellular sensitivity to MEK inhibitors (Figure 2A). Dose-response follow-up studies using the 3^rd^-generation MEK inhibitors trametinib (GSK1120212) and TAK-733 confirmed sensitivity to MEK inhibition (Figure 2B). *In vivo* treatment of M120903 was performed on ten mice, five of which were treated with 0.3 mg/kg trametinib twice daily by oral gavage and five received vehicle. In this setting, trametinib suppressed both subcutaneous growth and plasma S100B levels (Figure 2C-D and Supplemental Figure 3B) and extended the time for the mice to achieve a tumor size that required ethical sacrifice (Figure 2E). Immunohistochemical analyses for the apoptosis marker cleaved caspase-3 indicated that trametinib-treated M120903 tumor cells died by apoptosis (Supplemental Figure S3C). However, despite these promising data, no active MEK inhibitor trial was available for the patient with *NRAS* mutations at Sahlgrenska University Hospital. Therefore, the patient was treated with standard therapies when progression to stage IV was detected.

**Figure 3:**
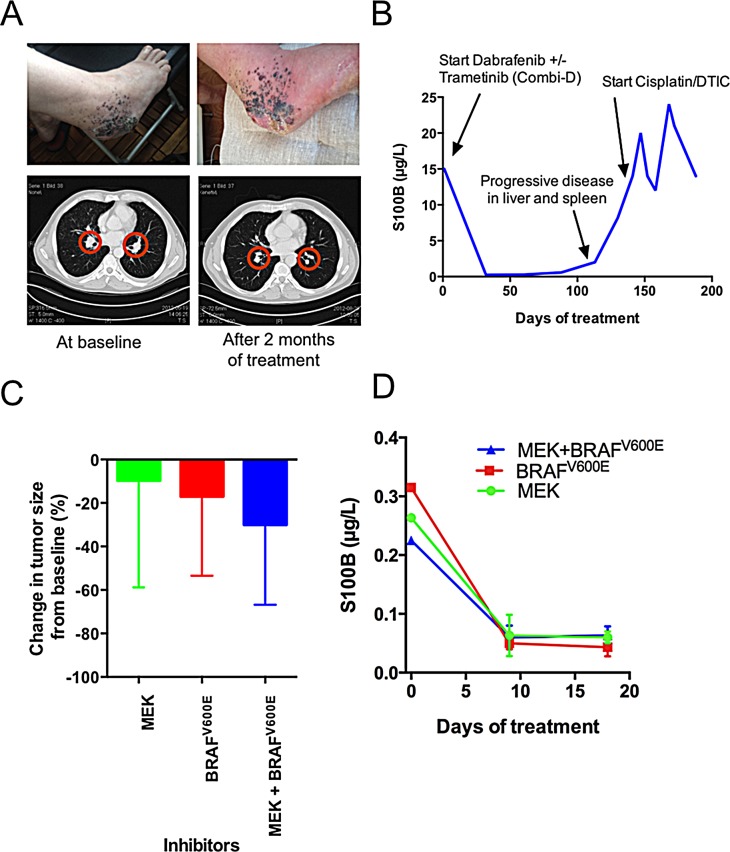
The responses of Case 2 (patient M120521A) to BRAF inhibitors, MEK inhibitors or both were similar in the patient and the PDX model (A) *Top* A photo provided by the patient showing responses at the primary site. *Bottom* CT scan showing the size reduction of lung metastases. (B) Plasma S100B levels during the treatment of M1201521A with BRAF inhibitors, MEK inhibitors or both. (C) The responses of the nine PDXes when treated with BRAF inhibitor, MEK inhibitor or both (n=3). Tumors were grown to 200 mm^3^ before treatment, and growth was monitored with a caliper. Shown are averagre size changes ± standard deviation. (D) Plasma S100B levels during treatment of the M1201521A PDX model with BRAF inhibitors, MEK inhibitors or both. Shown is average plasma S100B levels ± standard deviation.

The second case description concerns a patient diagnosed with an acral malignant melanoma on his foot. Post-operatively, an aggressive metastatic pattern was observed locally that included engagement of the right inguinal lymph nodes. He underwent limb perfusion in March 2012, but a PET-CT in May revealed stage IV disease that included metastases in the lung, liver and bone. One of the subcutaneous metastases was used to create a PDX (M120521A). Because the biopsy revealed that the tumor carried a *BRAF* V600E mutation, the patient was invited to participate in a double-blind clinical trial in June 2012. Here, he received either a BRAF inhibitor or a BRAF inhibitor in combination with an MEK inhibitor. The clinical arm to which he was recruited has not yet been disclosed by this global phase III trial. Nevertheless, to assess the clinical usefulness of our platform, we randomized nine mice carrying this patient’s tumor into three treatment groups receiving vemurafenib (BRAF inhibitor), trametinib (MEK inhibitor) or a combination, mimicking the clinical trial design. The patient exhibited objective responses observed by CT (Figure 3A) as well as by a drop in plasma S100B levels (Figure 3B). Reassuringly, both the tumor size (Figure 3C) and plasma S100B levels (Figure 3D) were also decreased in the PDXes, although no significant difference in response was observed between the treatment groups.

**Figure 4:**
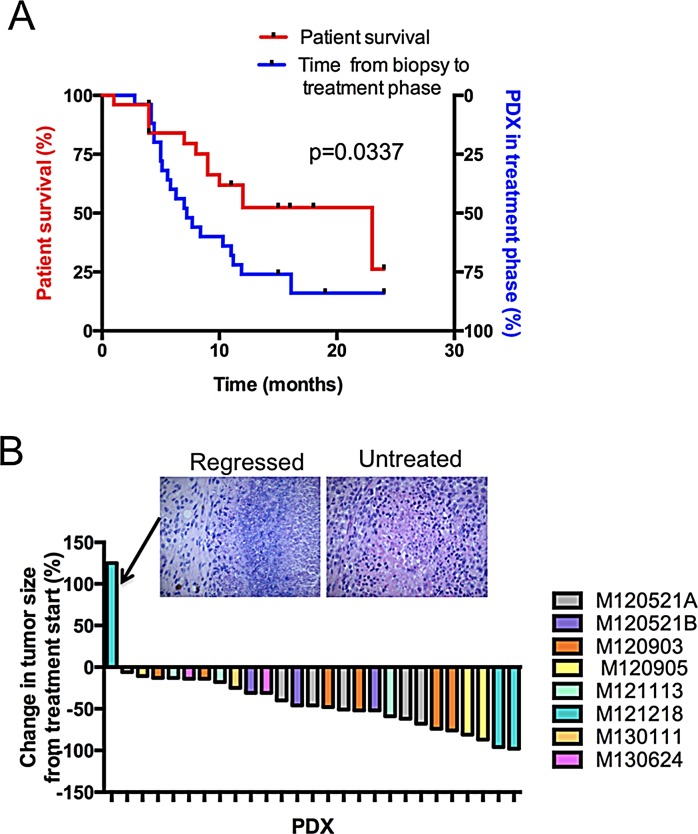
Treatment responses from the PDX platform can be generated before patient death (A) Kaplan-Meier plot comparing the time to death after biopsy of the included patients (left y-axis) with the time until the treatment phase of the PDX platform is reached (right y-axis). (B) Waterfall plot showing the treatment responses of a pilot pre-selection screen for a clinical trial of the MEK inhibitor trametinib. Note that one tumor did not appear to regress from baseline according to caliper measurement. Histological examination by a clinical pathologist demonstrated that this tumor had regressed and that the remaining mass consisted of fibrous tissue (inset).

To determine whether the described platform can be used to include patients to clinical trials, we surveyed the medical records to investigate whether patients were alive when the PDX models reached the treatment phase P3. As shown in the Kaplan-Meier plot (Figure 4A), the platform generally reached P3 faster before the corresponding patient had succumbed to the disease (P<0.05). This suggests that time does not preclude the use of PDX models as a pre-screening method in the recruitment phase of clinical trials. To assess the variability of response to therapies, PDX models from different patients were treated with MEK inhibitors. Most patients’ PDXes had tumors that exhibit sensitivity to MEK inhibitors (Figure 4B), which is in line with the known activation of the MAPK pathway in melanoma. Immunohistochemistry showed signs of regression, confirming that the tumors responded to the treatment, even if the tumor size was not reduced (Figure 4B and data not shown).

## DISCUSSION

Clinically useful biomarkers are often humoral markers, genetic markers or histochemicals staining, since they can be easily assessed in certified laboratories. Here, we describe an advanced platform used to identify treatments and/or clinical trials of new compounds where a biomarker of response is not yet known. The utility of similar *in vitro* or *in vivo* screens has been proposed previously [12-15]. Originally though, the use of these screens was limited by the lack of advances in tumor genomics, a lack of targeted therapeutics and low take-rates in the mouse strains used. Later, PDXes have been used to direct treatment responses in patients but success rates are not clear [10, 16]. Here we show that for melanoma, even in unselected and consecutively recruited patients, the frequency of patients from which PDX models were generated was high enough to make it clinically meaningful.

The clinical infrastructure of a University Hospital in a country with socialized medicine would normally be an obstacle for this type of translational research. Moreover, the cost per patient is significant, surpassing that of a PET-CT analysis. To circumvent these issues we suggest that the described platform will be most suitable in well-planned clinical trial, where a response in the PDX is an inclusion criteria [16], and in clinical research projects. Initiation of such trials is currently on the way. Given the high take-rate of melanoma in NOG mice [11], we postulate that PDX-driven clinical trials could be optimal for this patient group.

Reassuringly, the transplanted tumors exhibit a growth pattern similar to human melanoma (Figure 1). By expression profiling, we observed that PDXes were highly similar to melanoma from patients. To our knowledge, this is the first time melanoma PDXes have been compared with biopsy material directly from patients using RNAseq. Several investigators are moving away from the use of cell line xenografts in favor of PDXes [7, 8] and genetically engineered mouse models of cancer [17] in the pre-clinical phase of drug development. Our data demonstrate that PDXes are very similar to patient’s tumor, suggesting that the high rate of attrition observed with compounds in phase II may be lowered if these models are used in the future.

We also described two cases that demonstrate that the platform is experimentally feasible (Figure 2) and that treatment responses in PDXes are similar to the treatment response in the corresponding patient (Figure 3). However, we also voice some level of concern. First, *in vitro* cell cultures were not possible to generate from all tumor biopsies using standard cell culture conditions. However, given the similarity between the patient’s biopsies and PDXes, the platform is probably better off focusing on the use of PDXes. A second problem we had to tackle was that the initial take and growth of the tumors in different mice carrying the same tumor varied. This problem may be circumvented if tumor pieces were transplanted instead of single cells. We chose to use single-cell suspension in the transplants because the cells could be readily cryopreserved. Cryopreservation will be an added benefit if patients are included who have less advanced disease and may be cured by lymphadenectomy or adjuvant or immune therapies. Cells can be cryopreserved after P2 and re-initiated as PDXes only if the patient’s disease progresses.

Plasma S100B measurement was a robust method to assess therapy. Because personalized cancer treatment is expected to be based on low *n* data, we recommend using several methods of measuring therapeutic responses in PDX models. For melanoma, these methods include caliper measurements, measurements of a known biomarker, such as S100B or MIA, or immunohistochemical examination of treated tumors with H&E straining or antibodies directed against Ki67 and/or apoptosis markers.

For PDX-guided clinical treatments or inclusions into clinical trials to be useful, patients must provide biopsies early in the disease progression to allow sufficient time for the mice to develop tumors. We convincingly demonstrate in Figure 4 that this platform is possible to use even in consecutive patients that have not been selected based on gender, age and stage of metastatic melanoma. Although we demonstrate for the first time that there *is* time, focusing on early-stage metastatic melanoma or stage IV with oligo-metastatic disease may further improve the chances of including patients in PDX-driven clinical trials. Moreover, recent development of immune therapies, including adoptive T-cell transfer, vaccines and antibodies, has already demonstrated long-term survival of several melanoma patients [5]. It is likely that PDX-driven clinical trials will be initiated on a high proportion of patients where immune therapies have been partially successful, failed or deemed medically unsuitable. At that time, the fact that the PDX models are immunocompromised may be less of a concern.

We have recently demonstrated that inhibitors of MTH1 (which inhibit the growth of the M121218 PDX model [18]), Pim kinases [19], Aurora kinases [20] and Chk1 kinases [21] are promising anti-cancer drugs; however, because these drugs lack a biomarker of response, they will most likely require pre-selection of patients using a platform such as the one described here for the clinical trial to be successful. Indeed, even FDA-approved inhibitors appear to benefit from pre-selection because there is no known biomarker of response predicting which *NRAS*-mutant tumors respond to e.g. MEK inhibitors [22].

## MATERIALS AND METHODS

### Study design

The objective of the study was to determine if melanoma patient-derived xenografts were accurate models of the human disease, if they would respond to treatments known to cause tumor responses in patients and if these models could be developed fast enough to be used as pre-selection tools to guide the right patient to the right clinical trial. Thirty patients were recruited to the Department of Surgery where their tumors were excised and used to generate PDXes and when in excess, material for primary cell cultures and genetic and pathology analyses. If the tumors grew in mice (passage 1; P1), these were excised and subjected to serial transplantation. In P3, mice carrying the same PDX tumors were randomized to be treated with small molecule inhibitors via oral gavage. Treatment responses were monitored by caliper measurements of tumor size, measurement of serum levels of the melanoma marker S100B, routine histology and immunohistochemical methods and by comparing survival of mice treated with compound or vehicle. Mice were sacrificed when they were moribund, defined as when they became listless and exhibited ruffled fur and exhibited weight loss. Tumor analyses, RNAseq, pathology examinations, and cell experiments were performed in a blinded fashion. Comparison between the time to death from the biopsy was taken and the time needed to generate P3 PDXes was performed post-hoc since *a priori* knowledge of the utility of the platform was uncertain.

### Patient sample processing

Tumor biopsies from 30 patients with a confirmed diagnosis of malignant melanoma were collected from consenting patients (Regional Human Ethics Board of Västra Götaland, Sweden, #288-12) undergoing surgical tumor resection at the Department of Surgery at Sahlgrenska University Hospital, Gothenburg, Sweden. Upon collection, the samples were mechanically dissociated with and filtered through a cell strainer. The cells were washed with and resuspended in RPMI-1640 media and used for patient-derived cell cultures and the generation of PDXes.

### Cell culture, drug screen and follow-up

To generate a patient-derived cell line, an aliquot of dispersed cells of a patient’s tumor biopsy was diluted in complete media (RPMI-1640 supplemented with 10% fetal bovine serum, glutamine and gentamycin) and cultured at 37°C with 5% CO_2_. Cells from a confluent 10-cm plate were plated in four 96-well plates and used in a drug screen. The results were confirmed using two MEK inhibitors (GSK1120212 [trametinib] and TAK-733; Selleck Chem) to assess dose response sensitivity in a 96-well plate.

### PDXes and mouse treatments

All animal experiments were performed in accordance with E.U. directive 2010/63 (regional animal ethics committee of Gothenburg approval #287/289-12 and #36-2014). An aliquot of dispersed patient cells was mixed with an equal volume of Matrigel and injected subcutaneously into the flanks of immunocompromised, non-obese severe combined immune deficient interleukin-2 chain receptor γ knockout mice (NOG mice; Taconic, Denmark) to form xenografts. First-passage PDXes were passaged twice until they achieved 80-100 mm^3^ in the treatment phase. The mice were treated 5 days per week for a minimum of three weeks with 0.3 mg/kg trametinib (Selleck Chem) twice daily, 120 mg/kg vemurafenib (Zelboraf, a Roche product purchased at the hospital pharmacy) twice daily or both trametinib/vemurafenib twice daily. Once a week, tumor sizes were measured with a caliper. Before treatment, ten days after the start of treatment and at the end of the experiment, blood samples were drawn from the hind leg vein (vena saphena). Plasma levels of human S100B were measured with an ELISA kit (Abnova, Taiwan).

### Mutation and gene signature analyses

Mutation analyses were performed by three methods. First, the samples were genotyped at the Molecular Pathology Laboratory (KMP) at Sahlgrenska University Hospital by Sanger sequencing for *BRAF* mutation status as part of clinical routine. Second, allele-specific PCR was performed on small tumor pieces that had been lysed by overnight incubation in DirectPCR lysis buffer (Viagen) supplemented with proteinase K at 55 ºC. The primers directed against the mutant and wild-type forms of *NRAS* or *BRAF* have been described previously [23]. Third, next-generation sequencing (exome and/or RNAseq) was performed on DNA and/or RNA prepared from patient or xenograft biopsies using kits from Macherey-Nagel. Illumina sequencing was performed at the Genomics Core facility (exomes) at the University of Gothenburg or at BGI China (RNASeq) using Agilent capture kits. Variants were identified with the GATK package [24] and annotated with Annovar [25].

### Statistical statement

Statistical analyses were performed using GraphPad Prism 5. Two-sided t tests were performed, and the data are presented as the mean (± standard deviation) unless otherwise stated. Survival curve analysis for *in vivo* experiments was performed using the log-rank (Mantel-Cox) test. Statistical significance was set at P less than .05 (two-sided).

## CONCLUSION

The growth rate, characteristics and treatment response of melanoma patient-derived xenografts favor the use of these models as a pre-selection tool to guide the right patient to the right clinical trial

## SUPPLEMENTARY FIGURES AND TABLES


