# Supplementary material for: Melanoma patient-derived xenografts accurately model the disease and develop fast enough to guide treatment decisions
Source: Oncotarget. 2014 Sep 8;5(20):9609–18. doi: 10.18632/oncotarget.2445 (PMC4259423; doi:10.18632/oncotarget.2445)
Supplement: Supplementary file 1 [file oncotarget-05-9609-s001.pdf]

Melanoma patient-derived xenografts accurately models the disease and develop fast enough to guide treatment decisions

Supplementary Material

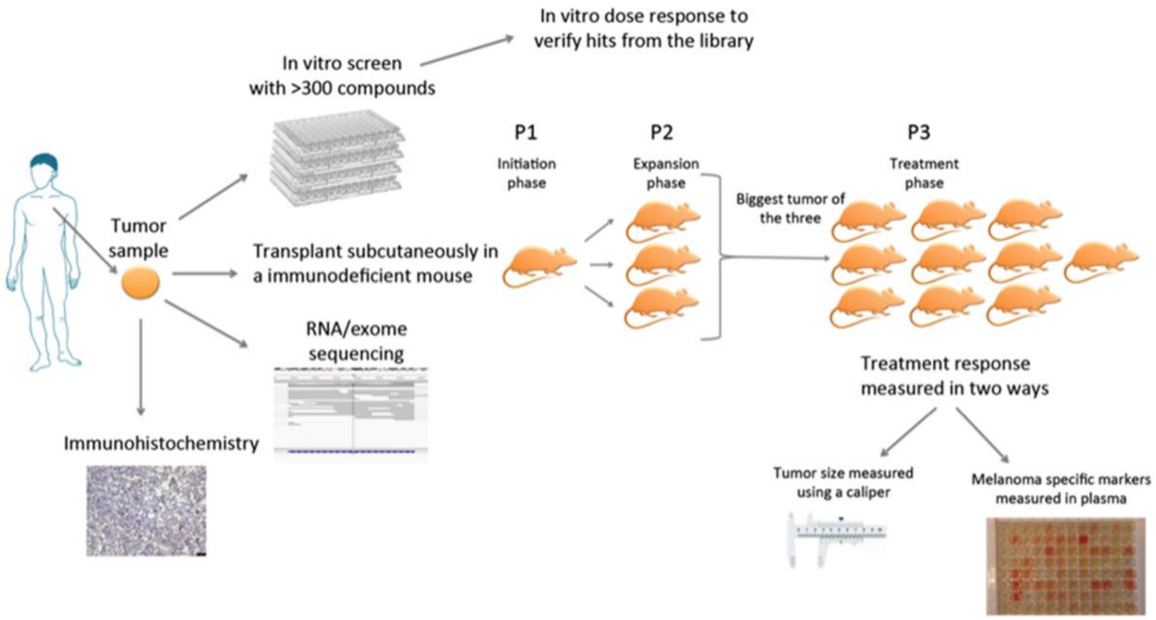

Figure S1: A platform of patient-derived tumor xenografts (PDXes) to identify new treatment options for metastatic melanoma. Schematic drawing of key steps in the process. Following resection, the tumors were dispersed into pieces, small enough to be drawn into a needle, and then injected subcutaneously onto the flank of (NOG mice to form passage 1 (P1) PDXes. Some of the cells or tumor pieces are also used for genomics and to establish cultures for drug screens. Following the establishment of P1 PDXes, mice were sacrificed and their tumors were serially transplanted into three new mice (P2), the fastest growing tumor of which was serially transplanted into ten mice (P3). In P3, mice are treated with various compounds emanating from genetic analyses, drug screens or available drugs in clinical trials.

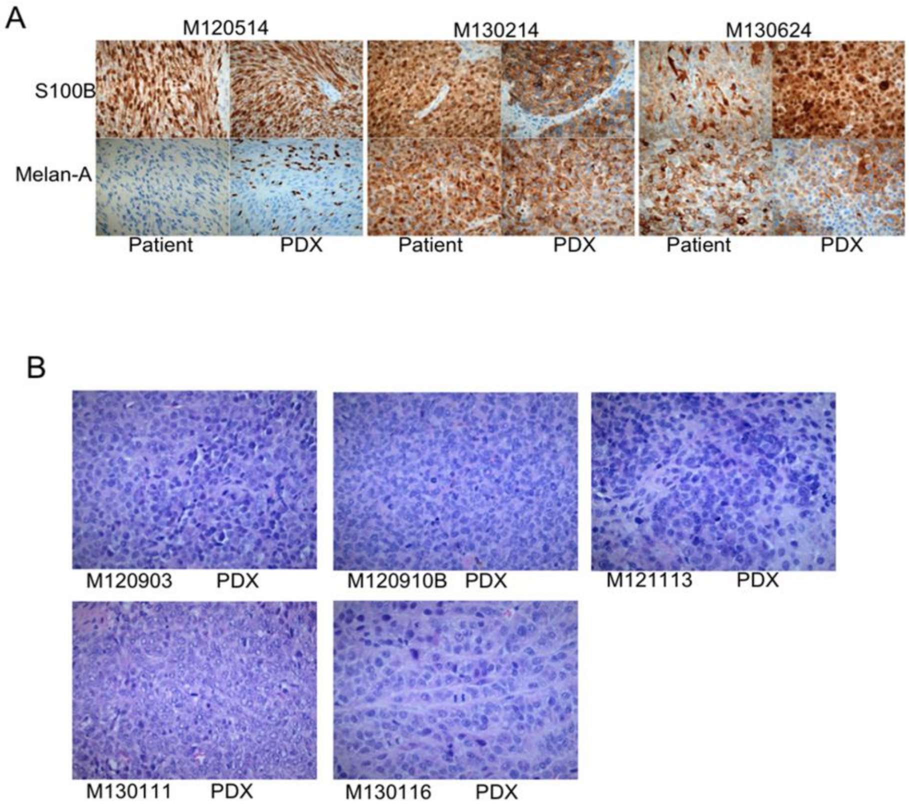

Figure S2: Melanoma PDXes resemble human melanoma. All samples were processed according to standard procedures at the clinical pathology lab. A clinical pathologist verified their similarity to human melanoma. (**A**) Immunohistochemical analyses of melanoma markers S100B, Melan-A and HMB-45 in paired patient and mouse tumors of three patients. (**B**) Representative images of H&E-stained formalin-fixed tumors from different PDX-models. Solid nests of tumor cells are surrounded by a delicate fibrovascular stroma. Numerous mitotic figures indicate rapid tumor proliferation.

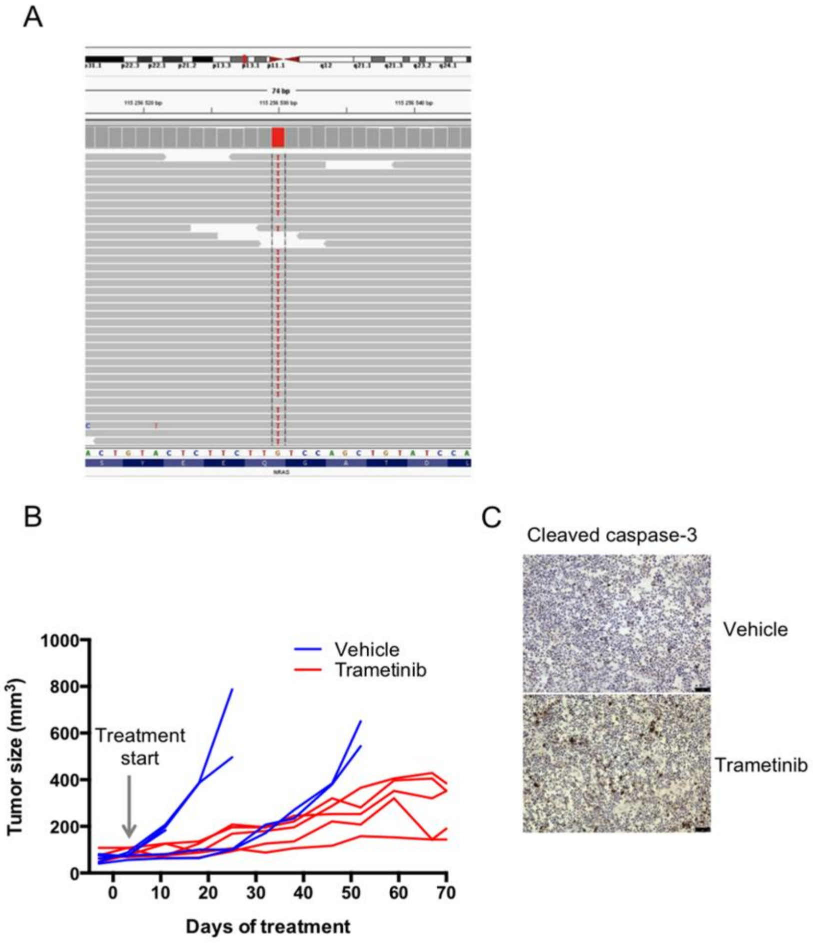

Figure S3: Trametinib has anti-tumor activity in PDX mice carrying the M120903 melanoma. (**A**) IGV screenshot showing the alignment between exome sequencing-generated reads and the human genome. A mutant nucleotide is shown in red. (**B**) Ten P3 PDXes were randomized to receive 0.3 mg/kg trametinib twice daily for five days a week by oral gavage or vehicle. Tumor growth was measured with a caliper. Shown here is the growth pattern of individual mice. Combined data, where day 0 is set as the timepoint when the tumors started to increase in size for two consecutive weeks, is shown in Figure 2C. (**C**) In a separate cohort of mice, three weeks after subcutaneous injection, palpable tumors were detected in the same PDX model. Mice were treated with vehicle or trametinib and tumors were harvested 6 h after the last injection. They were processed for immunohistochemistry using an apoptosis marker (cleaved caspase-3).

Supplemental Table S1: List of the PDX model biopsies and patient material subjected to RNA sequencing analysis. The amount of raw sequencing reads of the *CDKN2A* mRNA is also presented. Less than ten raw reads suggested that the gene was deleted or silenced by methylation. P1-3= passage 1-3, PR= original patient biopsy

Patient ID	CDKN2A reads
M120511B-2 - P1	104
M120511B -2 PR	93
M120521A - P1	17
M120521B - PR	22
M120903 - P3	0
M120905 - P2	4
M120910B - P2	1
M120913 - P1	30
M120913 - P2	40
M121113 - P2	1
M121123 - P2	1
M121211 - P3	38
M121213B - P1	147
M121213B - P1	197
M121218 - P1	197
M121218 - P2	340
M121218 - P3	191
M121218 - PR	158
M130111 - P2	120
M130116 - P3	25
M130128B - P2	353
M130128B - PR	265
M130214 - P1	0
M130214 - PR	31
M130226 - PR	72
M130228 - PR	743
M130624 - P2	0
M130624 - P3	0
M131004 - PR	424
M131118 - PR	1823

Supplemental Table S2: *BRAF* and *NRAS* mutation status. The patient biopsies were genotyped with Sanger sequencing (KMP, the clinical molecular pathology lab), allele-specific PCR or targeted re-sequencing (exome or RNA).

	KMP	qPCR		Exome sequencing		RNA sequencing	
Patient	B-Raf	Braf status	Nras status	Braf status	Nras status	Braf status	Nras status
M120511B-2	mut	mut	wt	V600E	wt	V600E	wt
M120514	mut	mut	wt	V600E	wt	n/a	n/a
M120521A	mut	mut	wt	V600E	wt	V600E	wt
M120521B		wt	mut	wt	Q61K	wt	Q61K
M120903	wt	wt	mut	wt	Q61K	wt	Q61K
M120905		wt	wt	wt	wt	wt	wt
M120910	wt	wt	mut	wt	Q61K	wt	Q61K
M120913	wt	wt	wt			wt	wt
M121113	mut	mut	wt			V600E	wt
M121123	mut	mut	wt			V600E	wt
M121211		mut	wt			V600G, V600M	wt
M121213A	mut	mut	wt			n/a	n/a
M121218	mut	mut	wt			V600E, V600M	wt
M121221	wt	wt	wt			n/a	n/a
M130111		wt	wt			wt	wt
M130116	mut	mut	wt			V600E	wt
M130128A	wt	wt	mut			n/a	n/a
M130128B		wt	mut			wt	Q61K
M130204B		wt	mut			n/a	n/a
M130214	wt	wt	wt			wt	wt
M130226	mut	mut	wt			V600E	wt
M130228		wt	mut			wt	Q61K
M130624	mut	mut	wt			V600E	wt
M131004	mut	mut	wt			V600E	wt
M131118		wt	wt			wt	wt
M140117	wt	wt	mut			n/a	n/a
